# COVID-19 Risk Perception and Prevention Practices among High- and Low-Density Populations in Bangladesh: A Mixed-Methods Study

**DOI:** 10.3390/tropicalmed7120447

**Published:** 2022-12-19

**Authors:** Syed Moinuddin Satter, Kamal Ibne Amin Chowdhury, Refah Tamanna, Zarin Abdullah, S. M. Zafor Shafique, Md Saiful Islam, Nadia Ali Rimi, Muhammad Rashedul Alam, Arifa Nazneen, Mustafizur Rahman, Taufiqur Rahman Bhuiyan, Farzana Islam Khan, Mahbubur Rahman, A. S. M. Alamgir, Tahmina Shirin, Mahmudur Rahman, Firdausi Qadri, Meerjady Sabrina Flora, Sayera Banu

**Affiliations:** 1 Programme for Emerging Infections, Infectious Diseases Division, icddr,b, Dhaka 1212, Bangladesh; 2 School of Population Health, University of New South Wales, Sydney, NSW 1466, Australia; 3 Institute of Epidemiology, Disease Control and Research (IEDCR), Dhaka 1212, Bangladesh; 4 Global Health Development, EMPHNET, Dhaka 1212, Bangladesh

**Keywords:** socioeconomic status, risk perception, risk prevention practices, qualitative, COVID-19

## Abstract

We aimed to explore coronavirus disease 2019 (COVID-19) risk perception and prevention practices among people living in high- and low-population density areas in Dhaka, Bangladesh. A total of 623 patients with confirmed COVID-19 agreed to participate in the survey. Additionally, we purposively selected 14 participants from diverse economic and occupational groups and conducted qualitative interviews for them accordingly. Approximately 70% of the respondents had low socioeconomic status. Among the 623 respondents, 146 were from low-density areas, and 477 were from high-density areas. The findings showed that study participants perceived COVID-19 as a punishment from the Almighty, especially for non-Muslims, and were not concerned about its severity. They also believed that coronavirus would not survive in hot temperatures or negatively impact Bangladeshis. This study revealed that people were reluctant to undergo COVID-19 testing. Family members hid if anyone tested positive for COVID-19 or did not adhere to institutional isolation. The findings showed that participants were not concerned about COVID-19 and believed that coronavirus would not have a devastating impact on Bangladeshis; thus, they were reluctant to follow prevention measures and undergo testing. Tailored interventions for specific targeted groups would be relevant in mitigating the prevailing misconceptions.

## 1. Introduction

Novel coronavirus disease 2019 (COVID-19), caused by severe acute respiratory syndrome coronavirus-2 (SARS-CoV-2), has become a global public health concern [1]. On 11 March 2020, the World Health Organization declared the COVID-19 outbreak a global pandemic [2]. As of 27 July 2022, 572 million confirmed cases of SARS-CoV-2 infection had caused 6.39 million deaths worldwide [3]. This novel virus is transmitted person-to-person via droplets and aerosols [4]. Population density [5] along with socioeconomic and cultural factors play an essential role in disease transmission and mortality [6].

Many COVID-19-affected countries have implemented various preventive measures, including national and zonal lockdowns, social distancing recommendations, isolation and quarantine of patients and contacts, guidelines for wearing facemasks, and recommendations for frequent handwashing to combat the spread of the virus [7]. South Asia’s lower-middle-income countries have taken initiatives to curb the rapid transmission of the virus [8]. India enacted the “Janata curfew” on 22 March 2020, and a 21-day complete lockdown starting on 25 March 2020 [8]. The Government of Bangladesh declared a national lockdown between 26 March and 30 May 2020, in the form of general holidays [9]. The GoB restricted mass gatherings, implemented bans on passenger movement on roads, water, and rail, suspended international and domestic flights, closed schools and colleges, and shut businesses, except for critical businesses and services [9]. People were requested to stay at home and maintain social distancing [9].

Adherence to public health measures is affected by beliefs, attitudes, and risk perception [10,11]. In a study in India, it was found that 90% of the respondents had knowledge about the name and origin, mode of transmission, symptoms, and prevention control of the virus, and they maintained the recommended measures, such as staying at home, elbow sneezing, maintaining social distancing, and wearing masks. However, there was a lack of perception. Of the respondents, 33.9% perceived that eating garlic could not prevent COVID-19, and 37.9% believed that the breath-holding test could not diagnose COVID-19 [12].

In Bangladesh, the first confirmed case was reported on 8 March 2020 [8], and as of 5 October 2020, the highest reported cases (64%) and highest reported deaths (50%) were in the Dhaka division [13]. In locations with a high population density, we do not know how people perceive the risk of respiratory infection or the benefits of non-pharmaceutical interventions. This is crucial, as in Dhaka, approximately 6 lakh people live in high-density areas where almost 75% of households share one room [14,15]. Moreover, because of shared toilets and kitchens, common water sources, and a lack of education, people living in these areas are more likely to be exposed to this virus [15]. Our study aimed to explore risk perception and prevention practices among high- and low-density populations in Dhaka, Bangladesh, during the COVID-19 pandemic.

## 2. Materials and Methods

### 2.1. Study Sites, Design, and Sampling

From July to September 2020, a multidisciplinary team comprising social scientists and epidemiologists conducted a cross-sectional study in Dhaka, Bangladesh. The team selected six high-density areas and seven low-density areas of Dhaka City for evaluation. High-density areas were horizontally shared spaces, with more than five people living in a 9–12 by 6–8-foot room (according to one of our ongoing studies, PR-20005). Low-density areas were areas with high-rise buildings and apartments.

We located symptomatic and asymptomatic laboratory-confirmed index cases in the community through the “Transmission Dynamics of COVID-19 in Bangladesh” study (PR-20005). The contacts of these patients were traced for enrollment, data collection, and sample collection. If any of the cases reported having neighborhood contacts, the team validated them based on the operational definition of contacts (a person who experienced face-to-face contact within 1 m and for more than 15 min, including travel, gossip, tea stall activity, or direct physical contact) between 2 days before and 14 days after the onset of symptoms in a confirmed COVID-19 case. The team developed a list of contacts for each case and validated it using phone calls or in-person visits. For qualitative interviews, we selected participants from diverse economic and occupational groups. The influential and informative persons of selected communities (i.e., ward counselors, ward members, community leaders, members of community-based organizations, schoolteachers, and religious leaders) who kept detailed updates of ongoing activities in their communities were considered to be study participants.

We adopted the WHO First Few X Cases and Contacts (FFX) Protocol (Version: 2, Date: 10 February 2020) that guided the B1 form for our survey [16]. The team also developed, piloted, and revised the interview guidelines before administration. The field team, consisting of five social scientists, received training on the study design, data collection, participant enrollment, interviewing, recording, note-taking, and data transcription. The field team also had several years of experience working on emerging infections.

### 2.2. Data Collection Methods and Techniques

We asked each contact for written informed consent and enrolled those who agreed to participate in the study. Survey interviews (conducted face-to-face or by mobile phone, depending on the respondent’s preference) were conducted to collect information on socioeconomic status, water safety, sanitation and hygiene (WASH) practices, and behavioral patterns related to coronavirus.

Through in-depth interviews (IDIs), we collected information on participants’ perceived understanding of COVID-19 and their knowledge of transmission pathways, their infection prevention practices, perceived and real challenges in maintaining prevention practices, experiences regarding treatment facilities (if any), opinions on isolation and lockdown, the impact of social stigma due to infection (if observed or faced), and the impact of lockdown on them and their households. Each IDI lasted for an average of 60 min and was recorded using an audio recorder. One note-taker was assigned to take notes, document non-verbal responses, and ensure tape recording.

Kuppuswamy’s socioeconomic scale (SES) is the most widely used scale for urban populations. We used a score of 3–29. This scale was developed based on a composite score of the family head’s education, occupation, and monthly family income. It was classified as high, middle, or low SES (Table 1).

### 2.3. Data Processing and Analysis 

All categorical variables collected from the survey were summarized using frequencies and percentages. Continuous numeric variables using mean and standard deviation and variables without a normal distribution were presented as medians and interquartile ranges.

Tape-recorded discussions during the qualitative interviews were transcribed in Bengali. The accuracy and consistency of the data were ensured as the researchers cross-checked the transcripts of the interviews. 

We sought assistance from Colaizzi’s phenomenological analysis method [19] and analyzed the qualitative data. Two anthropologists reviewed the data separately and identified themes and sub-themes that were shared among all the authors for discussion and consensus. 

### 2.4. Patient and Public Involvement

The study participants or associated persons were not involved in the design, conduct, reporting, or dissemination plans of this study.

### 2.5. Ethics Statement

The Institutional Review Board of icddr,b (PR-20066) reviewed and approved the study protocol. The Bill and Melinda Gates Foundation (BMGF) reviewed and relied on the IRB approval of icddr,b.

## 3. Results

### 3.1. Survey Results 

Among the 623 respondents who participated in our survey, 146 (23%) were from low-density areas and 477 (77%) were from high-density areas. A total of 288 (46%) were males and 335 (54%) were females. The mean age was 28.54 years, with a standard deviation of 15.24. A total of 238 respondents (38%) reported having completed primary education, 180 (29%) had completed secondary education, 44 (7%) had higher secondary education, and 161 (26%) had no institutional education. A total of 157 (25%) were service holders, 97 (17%) were dependent on daily wages for their livelihood, 52 (8%) ran small-scale businesses in their locality, 34 (5%) were unemployed, 157 (25%) were housewives, and 126 (20%) were students (Table 2).

#### 3.1.1. Socioeconomic Status 

Three families (0.5%) had high socioeconomic status, 76 (12.2%) had upper-middle socioeconomic status, 110 (17.7%) had lower status, 411 (66%) had poor socioeconomic status, and 23 (3.7%) had extremely poor socioeconomic status (Table 3).

#### 3.1.2. Water, Sanitation, and Hygiene (WASH) Access, Behavior, and Practices

The proportion of respondents who reported the use of improved sanitation facilities was significantly higher among low-density contacts (LD vs. HD, 56% vs. 25%, *p* = 0.0001), while the perceived importance of handwashing after urination and defecation and before eating was significantly lower among low-density contacts (LD vs. HD, 43% vs. 81%, *p* = 0.001) (LD vs. HD, 51% vs. 90%, *p* = 0.001) (Table 4).

Cleaning their clothing after coming home from outside every day was found to be significantly higher among high-density contacts (LD vs. HD, 60% vs. 73%, *p* = 0.02), while social distancing maintained by low-density contacts was significantly higher (LD vs. HD, 70% vs. 54%, *p* = 0.03) (Table 5).

### 3.2. Findings of Anthropological Exploration

Fourteen individuals (10 males, three females, and one transgender individual) participated in the qualitative study. Six of them were from high-density areas and eight were from low-density areas (Table 6). 

Among them, seven reported running small-scale businesses in their locality; three were service holders, one was a school teacher, and one was unemployed. The other participant was a health worker who had good acceptance in the community. Moreover, among these participants, one was a ward member who had an active influence on the community through various social activities during the lockdown period. Additionally, two were social workers and community leaders. The mean age of the participants was 38 years (range, 26–48 years). Five had a graduate degree, one had received higher secondary-level education, two had received secondary education, five had received primary-level education, and one did not have any institutional education. The religious background of all participants was Islam.

### 3.3. Risk Perception

#### 3.3.1. Beliefs in Supernatural Power 

The participants shared a common belief that the Almighty had created the coronavirus. Participants with limited or no institutional education did not consider COVID-19 a disease; instead, they believed that it was a punishment from the Almighty. Participants shared a firm belief that, since Bangladesh was a Muslim country, most people living there followed the Islamic ideology and Islamic-prohibited deeds were restricted there; therefore, the virus would not infect the people of Bangladesh.

The participants also believed that the coronavirus would infect non-Muslim people. Despite using the term “non-Muslim,” they specified the population as those who eat snakes, frogs, and scorpions. One participant from a low-density site or community stated that during the initial stage of COVID-19, there was a widespread belief in their community that COVID-19 would not enter a Muslim country. He also expressed that community members had a firm belief in Huzur’s (the mosque’s Imam) words. They did not want to maintain social distancing and protective measures following Huzur’s statements, as initially, Huzur mentioned that coronavirus would not enter a Muslim country and that Muslim people would not be affected by coronavirus. 

A 27-year-old male participant who was a service holder from a low-density area stated the following:

*“I am not against Huzur. However, the first mistake we made was a prevailing conception that Muslim people will not be infected by corona. Those who eat snakes, frogs, and scorpions will be infected. Besides, maintaining lockdown and restrictions were hampered because people obey Huzur’s words ten times more than regulation!”*

The participants also perceived that coronavirus was first reported in China during the winter season. As it was summer in Bangladesh at the time of the interview, they believed that the coronavirus would not survive or be transmitted.

A 33-year-old female participant who was a social worker from a high-density area stated,

*“If it could do anything, then there would have been a procession of corpses.”*

Those who believed that coronavirus depended on God’s will were also unwilling to maintain social distancing and personal protective equipment. 

#### 3.3.2. The Reluctance to Maintain Preventive Measures

Participants conveyed that there was an indifferent tendency regarding the use of protective measures. There was a lack of adherence to preventive measures, and community members were less inclined to maintain them. 

A 48-year-old male participant who ran a small business in a high-density area stated:

*“I saw rural people using a bamboo-made mask for domestic cows. Why would I wear such things that are used for cows?”*

People who belonged to low socioeconomic status groups and were engaged in services (those who ran a general store, shop in the bazaar, or tea stall) where they needed to deal with the general population were less inclined to wear masks. 

A 45-year-old male participant who was a small-scale businessman from a low-density area mentioned:

*“If I wear a mask all the time, customers do not understand properly what I was responding to them.”*

In low-density areas, people with low socioeconomic status are unwilling to maintain preventive measures. They perceived that as they lived from hand to mouth, God was more merciful to them, and, therefore, they would not be infected by the coronavirus. 

One participant in a high-density area stated that most of her neighbors preferred to die rather than maintain preventive measures. People with low socioeconomic status in low-density areas were unwilling to maintain social distancing or follow lockdowns. Middle- and lower-middle-class people were worried that if they did not earn a livelihood, they would die of hunger.

A 45-year-old male participant who ran a small-scale business in a low-density area stated the following:

*“We would rather die in corona but not out of hunger.”*

### 3.4. Perceived Reasons for Non-Adherence to Preventive Measures

#### 3.4.1. Financial Insolvency

Participants stated that financial constraints hindered the maintenance of protective measures. One participant said that buying masks and sanitizing hands with soap were beyond their affordability. One participant from a high-density area stated that he needed to think several times before buying a mask because a mask would cost at least BDT 15 (USD 0.2), which was expensive for him. 

Participants also mentioned their struggle to maintain isolation, even if their families had any patients who tested positive for COVID-19. All participants from high-density areas reported living in a single room with their families. They could not afford multiple rooms or spacious houses. Therefore, if any family members tested COVID-19-positive, they were unable to maintain isolation. 

A 40-year-old male participant, a small-scale businessman from a high-density area, stated:

*“I, along with my four family members, live in a single room. My neighbors as well as most of the families in our community, live in 10 feet by 10 feet single room where 6–9 members are living along.”*

According to the participants, community members were unwilling to undergo the COVID-19 diagnosis test because of their financial hardship.

A 26-year-old male participant who was unemployed and from a high-density area stated the following:

*“As it requires 3000–4000 taka (USD 35.71–47.61) for COVID-19 test, it is impossible for the lower-middle-class people to bear these expenses.”*

#### 3.4.2. Existing Rumors in the Community Regarding COVID-19 

Participants opined that the prevailing rumors might increase anxiety among community members and force them to maintain preventive measures. They perceived that if they were positive for COVID-19, they would be taken away to the hospital, would not be able to return home anymore, and would be killed with injections. According to some participants (3/14), the rumor was that if they became positive, they would either be kept in isolation or taken away by the police, and the whole area would be locked down. They would be detached from their family and friends and would not be able to earn money to continue their livelihoods. 

A 48-year-old male participant who ran a small-scale business from a low-density area mentioned:

*“The most common rumor in our community is that people think if someone tested positive for COVID-19, she/he would be taken away to Dhaka Medical College hospital and killed by pushing injection. We heard people are dying in hospitals for lack of treatment, oxygen, and food, etc.”*

### 3.5. Prevention Practices during COVID-19

Participants stated that government and non-government organizations disseminated preventive messages during the initial stage of COVID-19 and initiated restrictions, such as one-meter physical distancing and isolation. When these restrictions stopped, people became indifferent to maintaining social distancing, began roaming outdoors, and gathered for leisure time.

#### 3.5.1. Handwashing

Participants in low-density areas stated that during the lockdown period, people became habituated to washing their hands and were used to maintaining this seriously. People panicked, and they did this out of excitement (*Hujug*). One participant said that there were arrangements for washing hands at essential points, such as the marketplace, and that people had to practice handwashing. In addition, people wash their hands after returning home from outside. However, these practices gradually faded.

#### 3.5.2. Use of a Mask

One participant in the low-density area said that most people in his community were not inclined to wear masks unless there was a fear of police or community leaders reinforcing wearing them while going outside. He also said that some people perceived that wearing masks would spread more viruses. He opined that one of the reasons was illiteracy, and the other was religious influence. In the beginning, he noted that religious leaders told people that if they wore masks, they would be safe. However, later in mosques, *Wazz Mahfil* and *Boyan*, the *Huzurs* stated,

*“Nothing will happen. If God gives sickness, there will be nothing to do.”*

People were not inclined to wear masks initially, but later they realized this and prioritized them. A 26-year-old male participant who was unemployed and from a high-density area stated that 95% of the people were not self-conscious and less prone to wearing masks in his community. During the initial period of COVID-19, death and infection rates were broadcast on television as breaking news. Participants became tensed and panicked accordingly. However, when this briefing stopped, people started assuming that everything had returned to “normal.” He also added that only a limited number of people were still concerned, as the educational institutions remained closed, and when all these opened, people started thinking that everything was as expected. 

One participant in the high-density area said that in his community, most people had no educational background and were less inclined to accept the gruesomeness of the virus. He also stated that people between the ages of 40 and 50 were unwilling to maintain preventive practices. They just agreed during counseling but later did not maintain it. 

#### 3.5.3. Maintaining Social Distancing

One participant in the low-density area stated that people later realized the importance of social distancing. They were not serious about COVID-19 in the initial period and did not believe that the coronavirus would affect Bangladesh. He also added that people maintained social distancing during the lockdown period, and in some cases, they were forced to do so.

#### 3.5.4. Not going Outside the Home

A 27-year-old male participant who was a service holder from a low-density area stated that when there was a tense situation regarding the coronavirus, people were serious about it and tended to go out less. However, because they stayed home for a long time, people started feeling uncomfortable, and the rules were not appropriately maintained. 

Participants also mentioned that they used to go outside the home only during emergencies, such as buying rice, vegetables, and baby food, while wearing masks.

A 29-year-old female participant, who was a schoolteacher from a low-density area, mentioned:

*“I went outside for an important purpose, not for roaming aimlessly.”*

#### 3.5.5. Isolation of Infected People at Home

One participant in the low-density area stated that people belonging to the middle and lower-middle classes did not want to reveal whether they were COVID-19-positive because of an inferiority complex. He also added that the isolation of a person positive for COVID-19 was not appropriately maintained. 

A participant from a high-density area who was COVID-19-positive stated that she could not maintain proper isolation during that period. She shared a bed with her husband and her four-year-old daughter. She said that she did not have any other options; she neither had her own house nor the capability to rent a house outside this area. 

One participant in the high-density area shared a community incident: a ward counselor wanted to arrange a separate room for the isolation of 4–5 people who tested positive for COVID-19, but their family members would not allow them to live separately. The family members thought that the COVID-19-positive person would not be adequately cared for if they lived separately.

#### 3.5.6. Raising Awareness, Providing Financial and other Required Support 

All participants reported awareness-raising initiatives in both high- and low-density communities, such as distributing masks, setting up handwashing stations, distributing leaflets, spraying disinfectants, and raising awareness by motivating community members to maintain hygiene.

In high-density communities, several organizations such as Building Resources Across Communities (BRAC), Dushtha Sasthya Kendra (DSK), and other anonymous foreign initiatives helped people by providing food (rice, oil, and potatoes), protective equipment (masks and soaps), and financial aid so that people in the lower-middle-class could remain at home and did not need to go out to earn their livelihood. It was also reported that the solvent families of low-density communities provided food packages, including rice, oil, and onions, to their insolvent neighbors.

## 4. Discussion

This study explored the risk perceptions and prevention practices during the COVID-19 pandemic in low- and high-density areas. The findings showed that participants were not concerned about COVID-19 and believed that the coronavirus would not have a devastating impact on Bangladeshis. The participants highlighted that Almighty Allah would save Muslims. They also believed that Bangladesh’s warm weather would create a barrier to the widespread transmission of COVID-19. Protective measures were not accepted as practical or feasible. Substantial misinformation and rumors in the community regarding government containment strategies and day-to-day dissemination of death and infection rates through authentic electronic media of the government were reported. Moreover, this study revealed that people were reluctant to undergo COVID-19 testing. Family members hid information about being COVID-19-positive and avoided complying with institutional isolation, which has the potential for household transmission.

Participants’ prevention practice was influenced by their perception. They perceived that COVID-19 was a punishment from God. A study conducted in another Muslim country showed that 73.5% of Arab residents believed that COVID-19 was a dangerous disease [20]. In a study, researchers showed that people’s religious and ethical beliefs affect their coping mechanisms for disease and treatment regime [21]. Researchers also showed that people usually follow their religious coping behavior (e.g., faith in God, prayer, help, and strength from God) to deal with stressful situations.

Safe water, sanitation, and hygiene are required to protect against this virus [22]. The findings showed that most respondents consumed purified water for drinking and used sanitizers and soap after returning home from outside (Table 4). This may be due to government intervention. Although evidence of the effectiveness of face masks as a prevention measure [23] is still a topic of debate, a significant proportion (88%) of our study participants mentioned that they consistently used masks outdoors (Table 5). One study suggested that early public interest in facemasks may be an essential factor in controlling the COVID-19 epidemic on a population scale. Social distancing is regarded as the most effective measure for disease mitigation [24]. Most countries have focused on social distancing based on experiences gathered from China [25]. Participants from a previous study [26] believed that social distancing and the use of facemasks could break the chain of COVID-19 spread. However, according to our study participants, protective measures such as wearing a mask, sanitizing hands with soap, and maintaining social distancing were not accepted as practical and feasible. These findings are in line with a study conducted in Nepal [27], where the authors showed that the high population density in South Asia’s urban areas makes it difficult for people to maintain social distancing. A study conducted in Nepal [28] revealed a gap in knowledge regarding social distancing and quarantine; however, a positive perception of universal safety measures for COVID-19 has been reported. Another study [29] also shared participants’ poor knowledge of preventive measures.

Misinformation and rumors regarding government containment strategies, lockdowns, institutional isolation, and treatment management of patients admitted to hospitals during the early period of the pandemic were prevalent in communities. Similarly, a study conducted in India [30] reported gaps in the correct perception of knowledge and the propagation of myths and misconceptions. This finding suggests the need for educational programs to address misconceptions. Other studies [31,32,33,34] have also reported misconceptions regarding this disease. This study also found that community members did not trust the government’s daily announcements of deaths or infection rates. They perceived that the government announced an estimated number rather than an accurate one. Accurate information shared by the media plays a role in shaping people’s perceptions of the risk of COVID-19 transmission; a lack of accessibility to this information can serve as a barrier [35]. Studies conducted in India and northern Iraq have also reported the spread of fake news on social media [12,36]. This study also revealed that due to the financial hardship and misinformation prevalent in the community, people were reluctant to undergo COVID-19 testing.

There is available evidence that individuals change their behaviors, and increasingly rely on social media influencers, especially during the pandemic situation. However, one of the limitations of this study was that it was out of scope to share the relationship between social media usage and COVID-19. 

## 5. Conclusions

This study portrays the diverse perceptions of people belonging to different socioeconomic backgrounds. It also reveals that people’s practices are influenced by their attitudes and perceptions of disease and risk. In our study, we found that those who had negative and apathetic perceptions of the disease were less likely to maintain safety measures. Moreover, religious beliefs and issues were found to play a crucial role in driving people toward new practices. Our findings suggest the need for feasible and effective health education programs that include religious leaders and could be aimed at enhancing people’s disease-related knowledge, thereby helping them to perceive such diseases properly and maintain safe practices accordingly.

## Figures and Tables

**Table 1:** Modified Kuppuswamy’s Socioeconomic Scale [17,18].

	Score
Education	
*Professional Degree*	7
*Graduate*	6
*Diploma*	5
*Higher Secondary Certificate*	4
*Secondary School Certificate*	3
*Primary School Certificate*	2
*Illiterate*	1
Occupation	
*Profession*	10
*Self-employed*	6
*Clerical, shop-owner, farmer*	5
*Skilled worker*	4
*Semi-skilled worker or driver*	3
*Unskilled worker or labor or rickshaw puller*	2
*Unemployed*	1
Family income per month (in BDT)	
*≥60,001*	12
*30,001–60,000*	10
*15,001–30,000*	5
*12,001–15,000*	4
*9001–12,000*	3
*3001–9000*	2
*≤3000*	1
Socioeconomic class	
*Upper/High*	26–29
*Upper Middle*	16–25
*Lower Middle*	11–15
*Poor*	5–10
*Extreme poor or Below the poverty line*	0–4

**Table 2:** Distribution and comparison of demographic characteristics among infected and non-infected contacts.

Characteristic	Infected Contacts, (*n* = 74) *n* (%)	Uninfected Contacts, (*n* = 549) *n* (%)	*p*
Density			
*Low*	25 (33.8)	121 (22.0)	<0.05
*High*	49 (66.2)	428 (78.0)	
Age, years			
*<18*	17 (23.0)	162 (29.5)	>0.05
*18–25*	22 (29.7)	105 (19.1)	
*26–60*	32 (43.2)	268 (48.8)	
*>60*	3 (4.1)	14 (2.6)	
Sex			
*Male*	25 (33.8)	263 (47.9)	<0.05
*Female*	49 (66.2)	286 (52.1)	
Education			
*No education*	11 (14.9)	150 (27.3)	<0.05
*Primary*	37 (50.0)	201 (36.6)	
*Secondary*	23 (31.1)	157 (28.6)	
*Higher Secondary*	2 (2.7)	26 (4.7)	
*Graduate and above*	1 (1.4)	15 (2.7)	
Occupation			
*Service*	19 (25.7)	138 (25.1)	>0.05
*Business*	6 (8.1)	46 (8.4)	
*Self-employed (independent workers, employers)*	9 (12.2)	88 (16.0)	
*Dependent*	40 (54.1)	277 (50.5)	
Religion			
*Muslim*	73 (99.0)	545 (99.2)	>0.05
*Hindu*	1 (1.0)	4 (0.8)	
Household size (median, range)	4 (1–14)	4 (1–14)	
Household size			
*≤4 members*	54 (73.0)	355 (64.7)	>0.05
*>4 members*	20 (27.0)	194 (35.3)	
No. of bedrooms (median, range)	1 (1–3)	1 (1–5)	
Average size of bedroom, sft (median, range)	120 (30–180)	120 (30–400)	
Sharing bedroom	71 (95.9)	529 (96.4)	>0.05
No. of family members sharing one bedroom (median, range)	3 (2–7)	3 (1–20)	
Average monthly income, BDT	17,939	17,846	
Average monthly expenditure, BDT	15,202	15,214	

**Table 3:** Distribution of SES among neighborhood contacts in low-density and high-density areas.

Characteristic	Low-Density (*n* = 146) *n* (%)	High-Density (*n* = 477) *n* (%)	*p*
Upper/High	3 (2.1)	0 (0.0)	<0.05
Upper Middle	28 (19.2)	48 (10.1)	
Lower Middle	29 (19.9)	81 (17.0)	
Poor	83 (56.8)	328 (68.8)	
Extremely poor or Below the poverty line	3 (2.1)	20 (4.2)	

**Table 4:** Comparison of WASH practices among neighborhood contacts in low-density and high-density areas.

Characteristic	Low-Density (*n* = 146) *n* (%)	High-Density (*n* = 477) *n* (%)	*p*
Drinking water sources			
*Tube-well*	9 (6.2)	32 (6.7)	<0.05
*Supply*	118 (80.8)	424 (88.9)	
Drinks purified water	98 (67.1)	325 (68.1)	>0.05
Purification of water	77 (52.7)	206 (56.2)	>0.05
Actions are taken for purifying water			
*Boil*	74 (96.1)	240 (89.6)	>0.05
*Use a water filter/gravel/ceramic/sand*	1 (1.3)	18 (6.7)	
Water source for drinking looks clean	143 (97.9)	442 (92.7)	>0.05
Hand washing station at home	145 (99.3)	476 (99.8)	>0.05
Hand washing duration, seconds (median, range)	20 (4–600)	20 (3–200)	
Assumption on hand washing duration (median, range)	20 (3–600)	20 (0–200)	
Use of sanitizer and soap after coming back home	141 (96.6)	460 (96.4)	>0.05
Frequency of hand washing in a day			
*1–2 times*	11 (13.9)	15 (3.1)	<0.05
*3–4 times*	35 (44.3)	119 (24.9)	
*>4 times*	33 (41.8)	343 (71.9)	
Assumption on occasions important for hand washing *			
*Before eating*	75 (51.4)	428 (89.7)	<0.05
*Before feeding a child*	11 (7.5)	46 (9.6)	>0.05
*Before cooking /preparing/serving food*	28 (19.2)	148 (31.0)	<0.05
*After defecation/urination*	63 (43.2)	385 (80.7)	<0.05
*After cleaning a child that has defecated/changing nappies/washing diaper*	12 (8.2)	23 (4.8)	>0.05
Toilet facility			
*Improved sanitation facilities*	82 (56.2)	118 (24.7)	<0.05
*Shared sanitation facilities*	64 (43.8)	353 (74.0)	
*Unimproved sanitation facilities*	0 (0.0)	6 (1.3)	
No. of household members/toilet (median, range)	7 (1–212)	12 (1–100)	
Frequency of cleaning toilet per day (median, range)	0 (0–7)	0 (0–2)	
Frequency of cleaning toilet per week (median, range)	2 (0–21)	2 (0–30)	
Hand washing station availability	136 (93.2)	439 (92.0)	>0.05
Soap or detergent availability	142 (97.3)	464 (97.3)	>0.05
Surface of house/floor			
*Cement*	79 (100.0)	456 (95.6)	>0.05
*Other*	0 (0.0)	21 (4.4)	
Options for cleaning floor			
*Sweeping*	34 (43.0)	121 (25.4)	<0.05
*Mopping*	44 (55.7)	355 (74.4)	
Surface of yard			
*Cement*	76 (96.2)	337 (70.6)	<0.05
*Soil*	3 (3.8)	59 (12.4)	
Options for cleaning yard			
*Sweeping*	61 (77.2)	380 (84.3)	<0.05
*Mopping*	15 (19.0)	53 (11.8)	

* multiple responses.

**Table 5:** Comparison of behavioral change of neighborhood contacts in low-density and high-density areas.

Characteristic	Low-Density (*n* = 146) *n* (%)	High-Density (*n* = 477) *n* (%)	*p*
Infection			
*Uninfected contacts*	121 (82.9)	428 (89.7)	<0.05
*Infected contacts*	25 (17.1)	49 (10.3)	
Frequently touch face/eyes/nose	59 (40.4)	182 (38.2)	>0.05
Practices during coughing/sneezing			
*Cover face with hands/elbow before coughing or sneezing*	84 (57.5)	286 (60.0)	>0.05
*Cover face with tissue or handkerchief*	36 (24.7)	94 (19.7)	
*Nothing is done*	11 (7.5)	54 (11.3)	
*Others*	15 (10.3)	43 (9.0)	
Mask use outside every time	128 (87.7)	422 (88.5)	>0.05
Type of mask			
*Face mask/surgical single-use mask*	22 (31.9)	142 (31.8)	>0.05
*Cloth mask*	46 (66.7)	286 (64.0)	
Frequency of cleaning mask (times/day)			
*0*	22 (31.9)	186 (41.6)	
*1*	43 (62.3)	252 (56.4)	<0.05
*2*	4 (5.7)	9 (2.0)	
Difficulty wearing mask	65 (44.5)	218 (45.7)	>0.05
Cleaning of outside clothes everyday	87 (59.6)	350 (73.4)	<0.05
Social distancing maintained	102 (69.9)	257 (53.9)	<0.05
Difficult behavioral changes due to SARS CoV-2			
*Do not rub hands over face/eyes/nose*	26 (17.8)	39 (8.2)	<0.05
*Wear mask outside of home*	48 (32.9)	198 (41.5)	>0.05
*Cover face with elbow before coughing or sneezing*	18 (12.3)	46 (9.6)	>0.05
*Wash hands with soap/use sanitizer after coming home from outside*	12 (8.2)	27 (5.7)	>0.05
Perceived positive behavioral change can protect from COVID-19	127 (87.0)	397 (83.2)	>0.05

**Table 6:** Socio-demographic profile of the qualitative interviewees.

Characteristics	Frequency (*n*)	Percentage (%)
Gender *Male* *Female* *Transgender*	10 3 1	71.4 21.4 7.1
Age group (years) *21–30* *31–40* *41–50*	5 5 4	35.7 35.7 28.6
Marital status *Married* *Single*	12 2	85.7 14.3
Religion *Islam*	14	100.0
Educational level *Illiterate* *<Secondary* *Secondary* *>Secondary*	1 5 2 6	7.1 35.7 14.3 42.9
Occupation *Employed* *Unemployed* *Business*	6 1 7	42.9 7.1 50.0
Place of residence *High density* *Low-density*	6 8	42.9 57.1

## Data Availability

Data cannot be shared publicly because they are confidential. Data are available from the respective department of icddr,b for researchers who meet the criteria for access to confidential data.
